# Development of Caco-2 cells expressing four CYPs via a mammalian artificial chromosome

**DOI:** 10.1186/s12896-020-00637-8

**Published:** 2020-08-20

**Authors:** Yumi Ohta, Kanako Kazuki, Satoshi Abe, Mitsuo Oshimura, Kaoru Kobayashi, Yasuhiro Kazuki

**Affiliations:** 1 Division of Genome and Cellular Functions, Department of Molecular and Cellular Biology, School of Life Science, Faculty of Medicine, Tottori University, 86 Nishi-cho, Yonago, Tottori, 683-8503 Japan; 2 Chromosome Engineering Research Center (CERC), Tottori University, 86 Nishi-cho, Yonago, Tottori, 683-8503 Japan; 3 Trans Chromosomics, Inc, 86 Nishi-cho, Yonago, Tottori, 683-8503 Japan; 4 Laboratory of Biopharmaceutics, Meiji Pharmaceutical University, 2-522-1 Noshio, Kiyose, Tokyo, 204-8588 Japan

**Keywords:** Mammalian artificial chromosome, Chromosome transfer, Cytochrome P450, Intestinal metabolism, Caco-2 cell

## Abstract

**Background:**

Oral administration is the most common way to deliver drugs to the systemic circulation or target organs. Orally administered drugs are absorbed in the intestine and metabolized in the intestine and liver. In the early stages of drug development, it is important to predict first-pass metabolism accurately to select candidate drugs with high bioavailability. The Caco-2 cell line derived from colorectal cancer is widely used as an intestinal model to assess drug membrane permeability. However, because the expression of major drug-metabolizing enzymes, such as cytochrome P450 (CYP), is extremely low in Caco-2 cells, it is difficult to predict intestinal metabolism, which is a significant factor in predicting oral drug bioavailability. Previously, we constructed a mouse artificial chromosome vector carrying the CYP (CYP2C9, CYP2C19, CYP2D6, and CYP3A4) and P450 oxidoreductase (POR) (4CYPs-MAC) genes and increased CYP expression and metabolic activity in HepG2 cells via transfer of this vector.

**Results:**

In the current study, to improve the Caco-2 cell assay model by taking metabolism into account, we attempted to increase CYP expression by transferring the 4CYPs-MAC into Caco-2 cells. The Caco-2 cells carrying the 4CYPs-MAC showed higher CYP mRNA expression and activity. In addition, high metabolic activity, availability for permeation test, and the potential to assess drug–drug interactions were confirmed.

**Conclusions:**

The established Caco-2 cells with the 4CYPs-MAC are expected to enable more accurate prediction of the absorption and metabolism in the human intestine than parental Caco-2 cells. The mammalian artificial chromosome vector system would provide useful models for drug development.

## Background

Bioavailability is an important area of concern in drug development. Poor oral bioavailability has led to drug withdrawal. Oral drug bioavailability is often limited by metabolizing enzymes and efflux transporters in the gut [1]. The Caco-2 cell line derived from human colon carcinoma is a commonly used model for estimating the intestinal absorption of new drug candidates. Although Caco-2 cells express a variety of efflux and uptake transporters, they have an absence or low levels of cytochrome P450 (CYP) isoforms, such as CYP3A4 and CYP2C, that are typically expressed in the human intestinal epithelium [2]. Therefore, Caco-2 cells are of limited use in evaluating the role of metabolism in intestinal absorption after oral administration. To predict the intestinal absorption of drugs more accurately, it is necessary to modify Caco-2 cells to increase their expression of CYP isoforms.

Some studies reported that CYP3A-mediated metabolism in Caco-2 cells was enhanced by transfection with both CYP3A4 and CYP oxidoreductase (POR) [3, 4], treatment with 1α,25-dihydroxyvitamin D_3_ [5, 6], or the combination of transfection with CYP3A4 and treatment with both sodium butyrate and 12-O-tetradecanoylphorbol-13-acetate [7]. In contrast, few studies aimed at enhancing multiple CYP isoforms in Caco-2 cells have been performed. Honkakoski and his collaborators created Caco-2 cell lines expressing nuclear receptors, pregnane X receptor and constitutive androstane receptor [8–10]. These nuclear receptors upregulated the expression of some CYP isoforms in Caco-2 cells, but CYP activities remained very low in the absence of 1α,25-dihydroxyvitamin D_3_. Therefore, a new approach is needed to introduce multiple CYP isoforms in Caco-2 cells.

Mammalian artificial chromosome (AC) vectors derived from native chromosomes have several advantages over conventional vectors [11]. ACs segregate freely from host chromosomes through a set of cell divisions, and are adapted to carry multiple target genes with a desired copy number and Mb-sized genomic regions with endogenous regulatory elements. Furthermore, ACs carrying genes of interest can be transferred into various target cell lines via microcell-mediated chromosome transfer (MMCT). Considering these advantages, ACs have been used to generate several model cells for pharmacokinetic and toxicokinetic studies [12].

Previously, a lack of CYP3A4 expression in the Caco-2 cell line was addressed through the introduction of exogenous CYP3A4 and POR, which is a coenzyme of CYPs, via a human artificial chromosome (HAC) vector derived from human chromosome 21 [3, 13]. The HAC carrying CYP3A4 and POR genes conferred sufficient CYP3A activity to parental Caco-2 cells to be useful for predicting the intestinal extraction ratio in humans. Recently, a mouse artificial chromosome (MAC) vector constructed from native mouse chromosome 11 was used to increase the activity of multiple CYPs in HepG2 cells, which are a liver cancer cell line typically exhibiting low CYP activity [14]. In this study, four CYP genes (CYP3A4, CYP2C9, CYP2C19, CYP2D6) and a POR gene were loaded on the MAC (4CYPs-MAC) and transferred to HepG2 cells (TC-HepG2) to make the cells more suitable as a model to evaluate drug–drug interactions (DDIs) and hepatotoxicity in the initial screening of candidate drugs [15]. The expression and activity of CYPs in TC-HepG2 were comparable to those in human hepatocytes and this expression was sustained after a long culture period because of the stability of the MAC in human cells [16]. Regarding the assessment of DDIs, the activity of CYPs in TC-HepG2 was reduced in a concentration- and time-dependent manner by specific inhibitors, which reflects the conditions in primary human hepatocytes. Furthermore, metabolic toxicity of aflatoxin B1, which is converted to its active metabolite via CYP3A4 and exerts hepatotoxicity through DNA damage [17], was clearly recapitulated in TC-HepG2 cells rather than parental HepG2 cells. This study suggested that TC-HepG2 can provide a useful model to assess not only hepatic metabolism but also CYP-mediated hepatotoxicity during the early stages of drug development and the system using the MAC can improve the existing cell-based model.

In the current study, we aimed to utilize previously constructed 4CYPs-MAC to generate a novel Caco-2 cell line with increased activity of multiple major CYPs. The 4CYPs-MAC was transferred to Caco-2 cells via MMCT to establish Caco-2 cells carrying the 4CYPs-MAC, and the Caco2 4CYPs-MAC cells were examined to determine whether they exhibited sufficient CYP activity for use in initial drug screening.

## Results

### MMCT and analyses of acquired clones

**Fig. 1:**
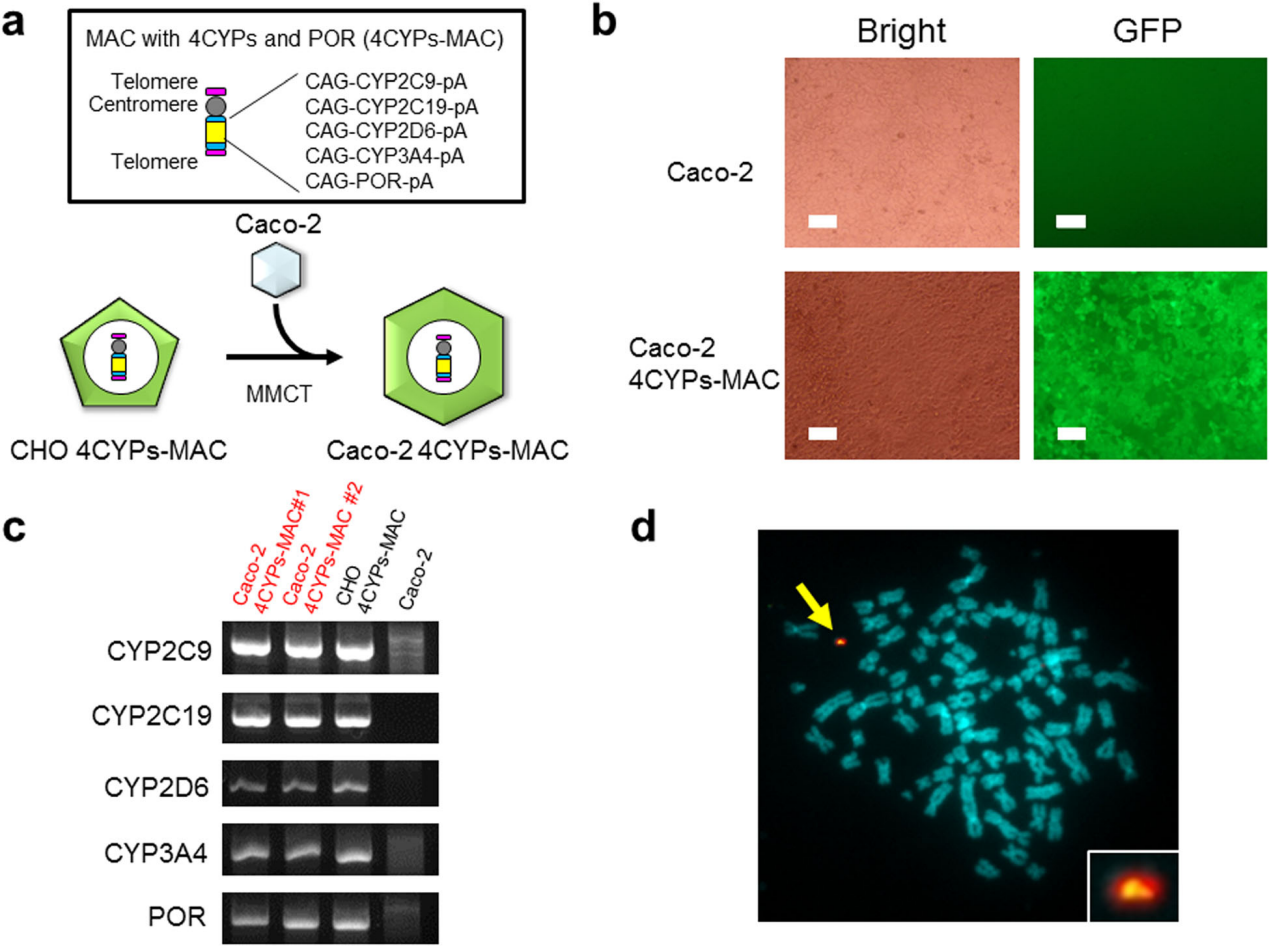
Introduction of the 4CYPs-MAC into Caco-2 cells. **a** Transfer of the 4CYPs-MAC into Caco-2 cells. The structure of the MAC carrying four CYPs and POR is shown at the top. A CAG promoter was placed upstream of each gene. A schematic view of the transfer of the 4CYPs-MAC to Caco-2 cells is shown at the bottom. The 4CYPs-MAC was transferred from CHO cells to Caco-2 cells through the MMCT method. **b** An image of GFP fluorescence in parental Caco-2 cells and Caco-2 4CYPs-MAC cells. The GFP fluorescence indicates the presence of the 4CYPs-MAC in the Caco-2 cells. The white bars indicate a distance of 50 μm. **c** Results of genomic PCR analyses to detect four CYP and POR transgenes on the MAC in Caco-2 cells. Donor CHO cells and Caco-2 cells are positive and negative controls, respectively. **d** Images of FISH analyses of Caco-2 cells carrying the 4CYPs-MAC. Red and green signals indicate the MAC and transgenes, respectively. The arrow shows the 4CYPs-MAC and the inset shows an enlarged image of the 4CYPs-MAC

CHO cells carrying a MAC vector with CYP2C9, CYP2C19, CYP2D6, CYP3A4, POR, and GFP genes were prepared (4CYPs-MAC) (Fig. 1a). Using CHO cells as donor cells and Caco-2 cells as recipient cells, we attempted to generate Caco-2 cells carrying the 4CYPs-MAC via MMCT (Fig. 1a). After selection, four drug-resistant GFP-positive clones were obtained (Caco-2 4CYPs-MAC) (Fig. 1b). To examine whether the CYP and POR genes were introduced into the obtained clones, genomic PCR analyses were performed. CHO cells with the 4CYPs-MAC and Caco-2 cells were used as positive and negative controls, respectively. Consequently, a band of the desired size was observed for each primer set in the candidate clones (Fig. 1c). Next, a chromosome specimen was prepared from the acquired clones and FISH analysis was performed to check the karyotype. FISH analysis revealed that a single copy of the 4CYPs-MAC existed in the candidate clones (Fig. 1d).

**Fig. 2:**
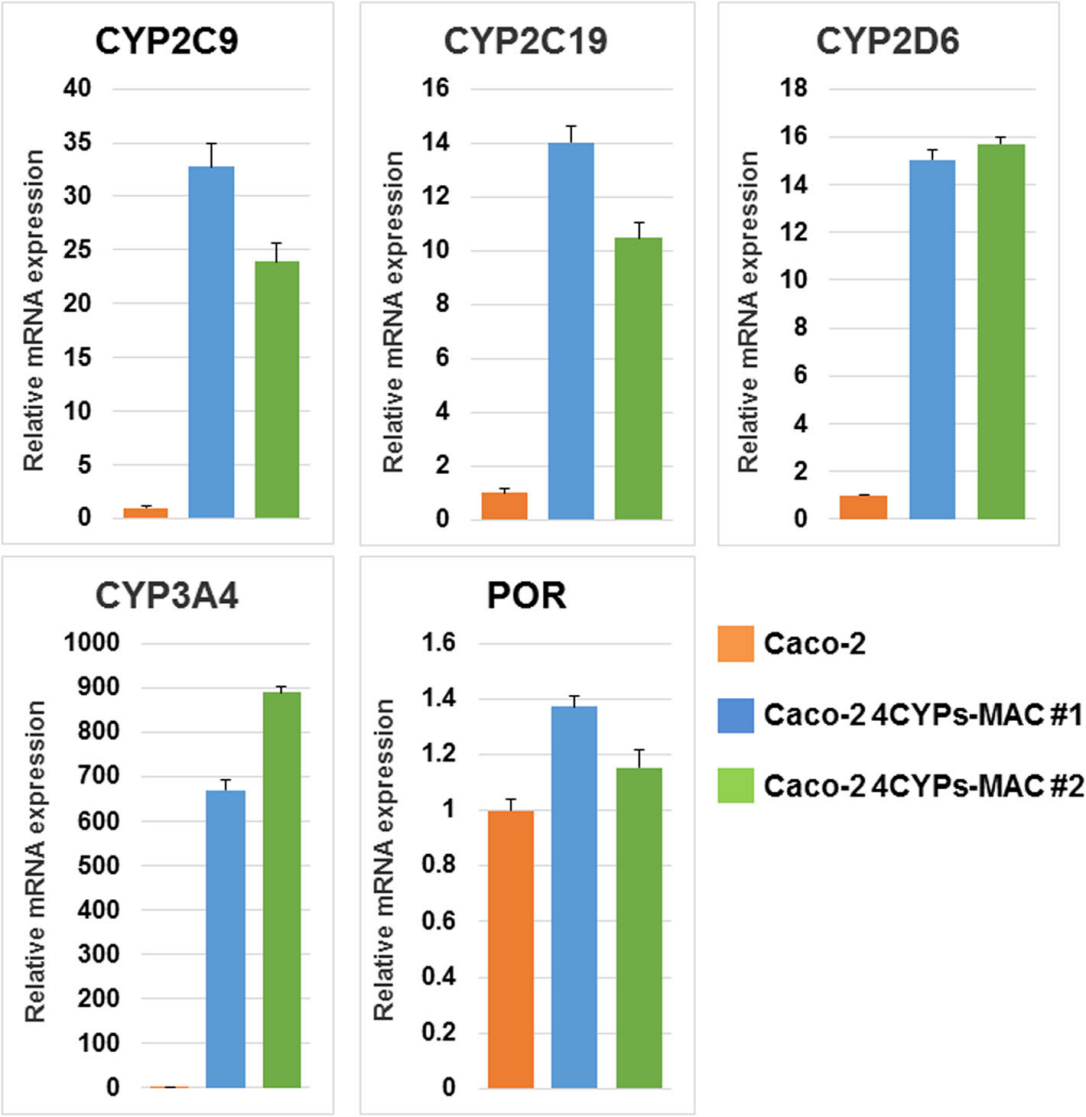
Gene expression analyses of Caco-2 cells with the 4CYPs-MAC. The expression levels of the four CYPs and POR in Caco-2 cells with the 4CYPs-MAC. The relative expression levels of the four CYPs and POR genes of the parental Caco-2 cells and acquired clones were analyzed through RT-qPCR. GAPDH was used for normalization (mean ± S.E., *n* = 3)

RT-qPCR analysis was performed to examine the mRNA expression level of the introduced CYPs and POR in the obtained clones. Compared with that in parental Caco-2 cells, the gene expression level was particularly high in the Caco-2 4CYPs-MAC #1 and Caco-2 4CYPs-MAC #2 clones (Fig. 2). Among the introduced genes, POR expression was only slightly enhanced in these clones. Basal expression of POR in parental Caco-2 cells is high, as observed in a previous study [3]. Caco-2 4CYPs-MAC #1 showed high expression of the majority of genes compared with Caco-2 4CYPs-MAC #2. The Caco-2 cell line consists of a heterogeneous population of cells [18]. Therefore, difference of the gene expression levels between obtained clones may partly depend on the population into which the 4CYPs-MAC has been introduced. Although the other two clones obtained by MMCT still showed higher expression levels than the parental Caco-2 cells, the level of increase was moderate. Therefore, we selected Caco-2 4CYPs-MAC #1 and Caco-2 4CYPs-MAC #2 clones for further analyses to evaluate the availability as an improved model system. These results suggest that we successfully transferred the 4CYPs-MAC to Caco-2 cells and the genes on the MAC were highly expressed.

### Monolayer formation of Caco-2 4CYPs-MAC cells

**Fig. 3:**
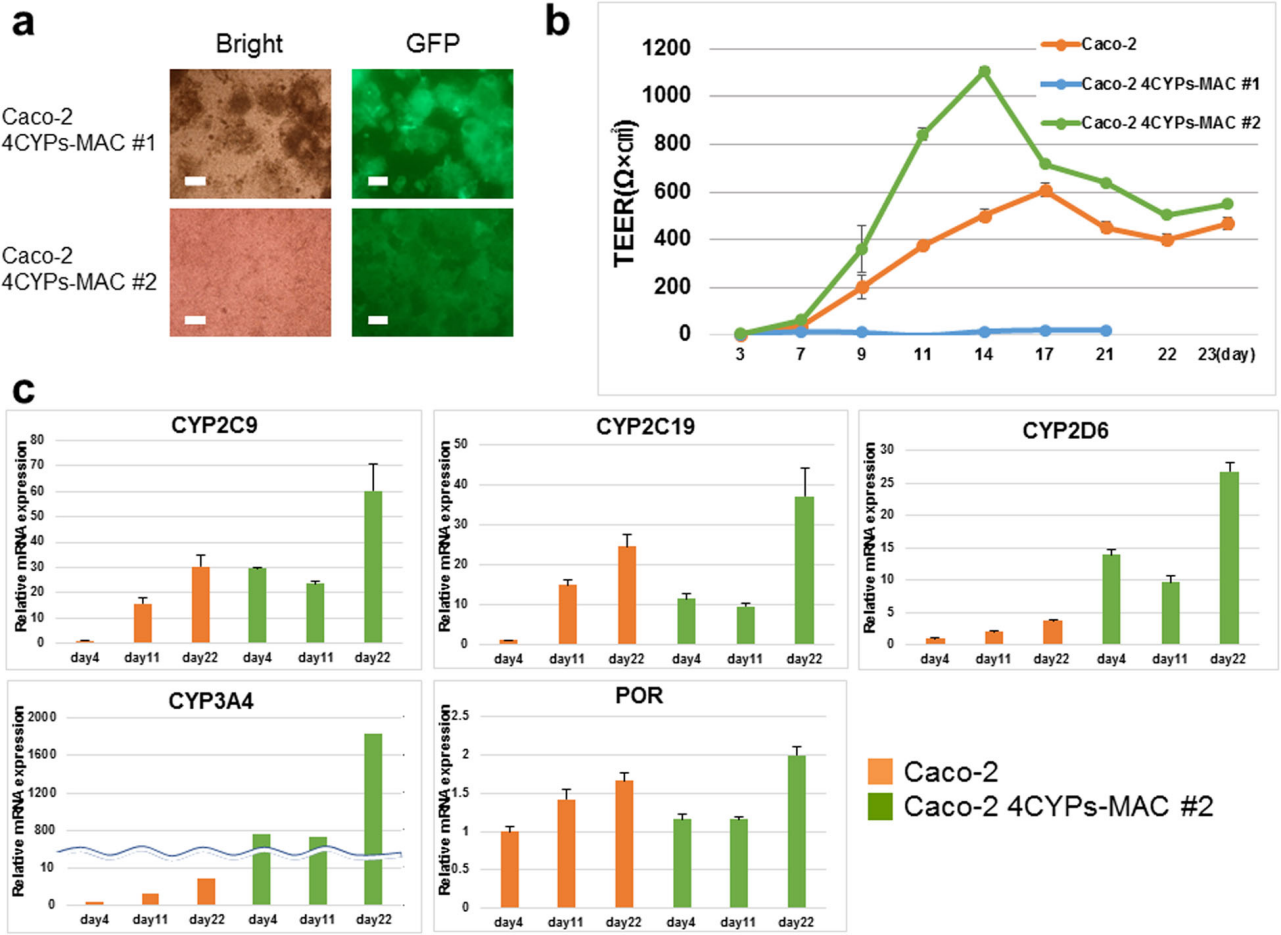
Monolayer formation assay. **a** Images of bright and GFP fluorescence from cells seeded on the membrane of a Millicell-24 plate. In Caco-2 4CYPs-MAC #1, cells did not spread throughout the membrane and did not form a cell layer, but in Caco-2 4CYPs-MAC #2, there were no gaps between cells, and they formed a cell layer. The white bars indicate a distance of 100 μm. **b** Transepithelial electrical resistance (TEER) values of Caco-2 cells, Caco-2 4CYPs-MAC #1 and Caco-2 4CYPs-MAC #2 cells. **c** Culture time-dependent change in gene expression. The relative expression level was evaluated in Caco-2 and Caco-2 4CYPs-MAC #2 (mean ± S.E., *n* = 3)

We seeded Caco-2 4CYPs-MAC #1 and Caco-2 4CYPs-MAC #2 cells at a concentration of 2.5 × 10^4^ cells/well in a Millicell 24-well cell culture insert plate. The Caco-2 4CYPs-MAC #2 cells spread across the entire membrane and formed a cell layer, while the Caco-2 4CYPs-MAC #1 cells did not spread and there were gaps in the cell layer (Fig. 3a). Caco-2 4CYPs-MAC #1 appeared to aggregate and form multiple layers rather than spread and form a single layer. It was reported that multilayered areas appeared in the cell population for late passage cells [19]. The TEER value was measured using a Millicell-ERS (Fig. 3b). The TEER value is an index of tight junction formation, and when the value is almost constant, it is considered that a cell layer has formed. With the exception of the Caco-2 4CYPs-MAC #1 clone, the Caco-2 cells and Caco-2 4CYPs-MAC #2 cells showed an increase in TEER value until it plateaued after 20 d. The Caco-2 cells and Caco-2 4CYPs-MAC #2 showed almost equivalent TEER values. Because monolayer formation is essential for the permeation test, the subsequent tests were performed using the Caco-2 4CYP-MAC #2 cells.

### Culture time-dependent change in gene expression

Total RNA was extracted from Caco-2 cells and Caco-2 4CYPs-MAC #2 cells on the 4th, 11th, and 22nd days after seeding. We compared the expression level of each gene on each day, and confirmed that the expression levels of the four CYPs and POR increased in both the Caco-2 cells and Caco-2 4CYPs-MAC #2 cells (Fig. 3c). The expression levels of all genes analyzed were significantly higher in the Caco-2 4CYPs-MAC #2 cells on the 22nd day than those in parental Caco-2 cells. The gene expression level increased depending on the culture time and the gene expression levels of the Caco-2 4CYPs-MAC #2 cells established in the current study were higher than those of parental Caco-2 cells. With the exceptions of CYP3A4 and CYP2D6, the expression levels in parental Caco-2 cells were higher in all cases until day 22. The parental Caco-2 cell line appears to have higher potential to enhance the expression of CYP2C9 and CYP2C19 during differentiation.

The expression levels in Caco-2 and Caco-2 4CYPs-MAC at day 22 were compared with those in human adult intestine (Additional file 1: Figure S1). In Caco-2 4CYPs-MAC #2, the expression levels of CYP2C9 and CYP2C19 were comparable and that of CYP2D6 was higher than in human adult intestine. Although CYP3A4 expression was significantly enhanced in Caco-2 4CYPs-MAC #2 compared with that in parental Caco-2 cells, the expression was still lower than in human adult intestine.

### CYP metabolic activity measurement

**Fig. 4:**
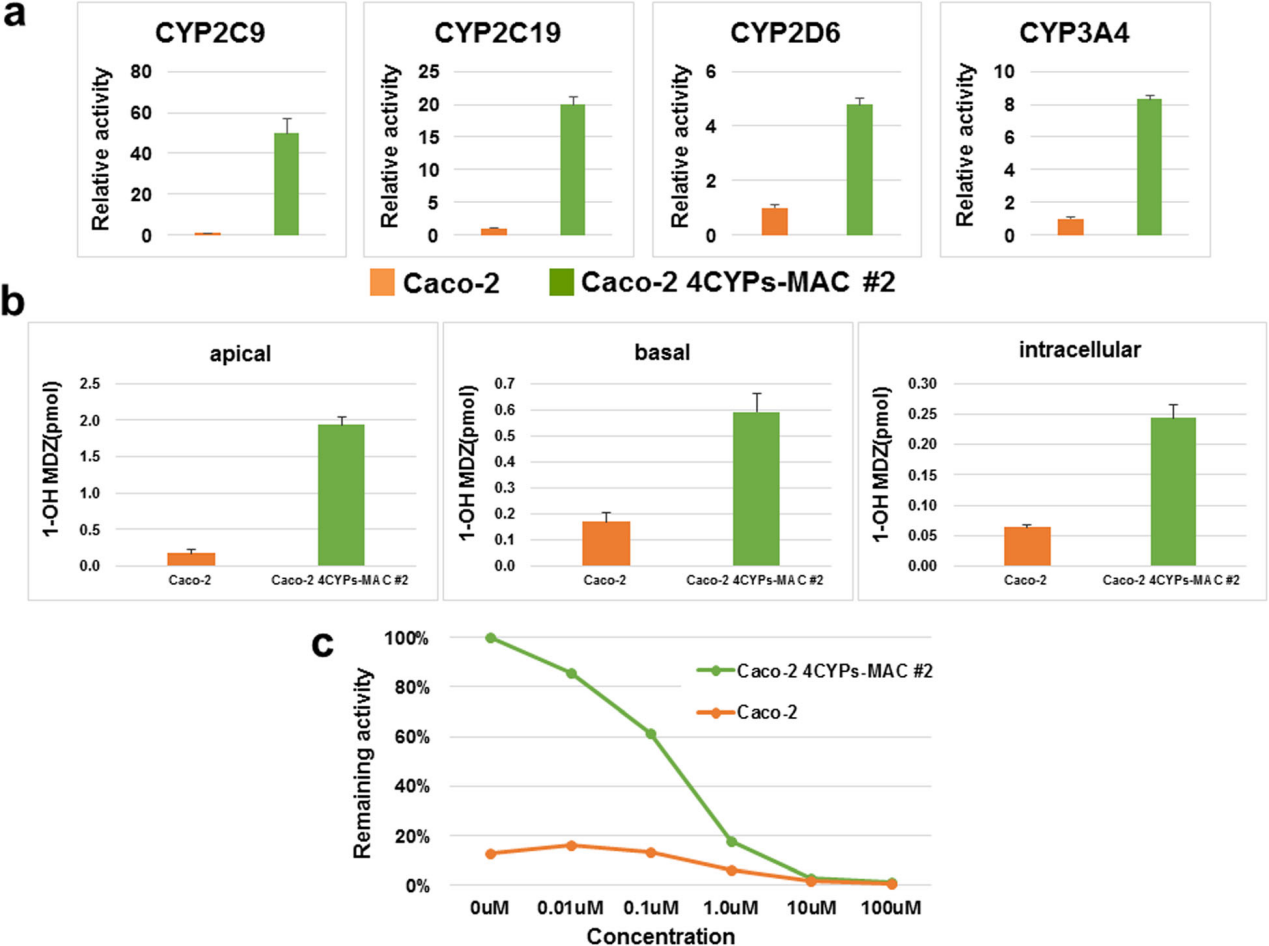
Activity of each CYP in the Caco-2 4CYPs-MAC cells. **a** The metabolic activity of each CYP in Caco-2 4CYPs-MAC cells. The relative activity for each CYP was measured by comparing the parental Caco-2 cells and the Caco-2 4CYPs-MAC #2 (mean ± S.E., n = 3). **b** Permeability test using MDZ. The permeability test was performed 23 d after seeding Caco-2 cells and Caco-2 4CYPs-MAC #2, whereby 3 μM MDZ was added to the apical side, and after 30 min, the apical, intracellular, and basal supernatants were collected. The 1′-OH MDZ in the supernatant was measured through LC-MS/MS. **c** CYP3A4 inhibition test. Ketoconazole, an inhibitor of CYP3A4, was added to the Caco-2 4CYPs-MAC #2 and incubated for 1 h. A luminescent substrate was measured to detect CYP3A4 activity with different concentrations of ketoconazole

A P450-Glo assay with each specific substrate was employed to measure the metabolic activity of CYPs in the Caco-2 4CYPs-MAC #2 cells, which had high gene expression levels, as confirmed through RT-qPCR analysis. The activities of all four CYPs were higher in the Caco-2 4CYPs-MAC #2 clone than in Caco-2 cells (Fig. 4a). The results indicate that the introduced 4CYPs-MAC expressed functional CYPs and increased the total activity of each CYP in the Caco-2 cells. The enhancements in the rates of metabolic activity of CYP2C9, CYP2C19, and CYP2D6 were generally correlated with those of mRNA expression. However, there was a gap between the enhancement of the rate of CYP3A4 mRNA expression and that of metabolic activity. This may have been because the parental Caco-2 originally had extremely low expression of CYP3A4.

### MDZ permeability test

A permeation test was conducted using midazolam (MDZ), a CYP3A substrate, to examine whether the cells reflected the behavior of small intestinal epithelial cells in terms of MDZ permeation. The penetration test was performed on day 23 after cell seeding when the TEER value plateaued. We measured the amount of 1′-OH MDZ in each of the donor side (apical), recipient side (basal), and intracellularly. The amounts of 1′-OH MDZ in all layers of the Caco-2 4CYPs-MAC #2 cells were higher than in those of Caco-2 cells (Fig. 4b). Moreover, ER was calculated using Eq. 1 and the results were 0.47 and 3.34% for Caco-2 and Caco-2 4CYPs-MAC #2, respectively. ER indicates the rate of metabolism during cell permeation. CYP3A4 was scarcely expressed in parental Caco-2 cells, so the ER value was extremely low. However, Caco-2 4CYPs-MAC #2 cells showed an ER of 3.34%, which was higher than in the Caco-2 cells, and MDZ was metabolized by CYP3A4 when passing through the cells.

### Inhibition test

To determine the availability of the established clone for the assessment of DDIs, we added ketoconazole, an inhibitor of CYP3A4, to the Caco-2 4CYPs-MAC #2 cells, and examined whether the metabolic activity was reduced. Ketoconazole at 0, 0.01, 0.1, 1.0, 10, and 100 μM was added and cells were incubated at 37 °C for 1 h followed by the measurement of metabolic activity. The metabolic activity of CYP3A4 decreased as the inhibitor concentration increased (Fig. 4c). The activity of CYP3A4 in Caco-2 4CYPs-MAC appeared to be sufficient for the inhibition test compared with that in parental Caco-2 cells. In addition to the permeation test for CYP3A4, inhibition of CYP3A4’s function by ketoconazole in Caco-2 4CYPs-MAC cells was also confirmed. This suggests that the established cells could be used for DDI testing of drugs that are substrates of CYP3A4. Therefore, the Caco-2 4CYPs-MAC cells more accurately reflect the behavior of CYP3A4 substrates in human epithelial cells than parental Caco-2 cells.

## Discussion

In the current study, we introduced four CYPs and POR into Caco-2 cells to increase their drug metabolic abilities, which are typically low. This study was intended to establish a better human cell model to more precisely evaluate the behavior of drugs in the small intestine. The 4CYPs-MAC was successfully introduced into the Caco-2 cells and successfully increased CYP activity.

The gene expression and activity of CYPs in TC-HepG2 carrying the 4CYPs-MAC are either comparable to or higher than those in primary human hepatocytes [15]. In this study, in contrast to the other CYPs, CYP3A4 mRNA expression was still low in Caco-2 carrying the 4CYPs-MAC compared with the level in human adult intestine, despite the significant enhancement of mRNA expression. Regarding the genes on the 4CYPs-MAC, each is present as a single copy. Because the nature of gene regulation is supposed to differ between HepG2 and Caco-2, changing copy number of the CYP3A4 gene may further optimize the expression profile of Caco-2 cells to that of human intestine.

The established Caco-2 4CYPs-MAC cells with particularly high gene expression showed high activity in all CYPs. In the future, we will conduct metabolic tests, inhibition tests, and permeation tests using drugs that are substrates for other CYPs, and investigate whether the Caco-2 4CYPs-MAC #2 cells reflect the behavior of drugs in small intestinal epithelial cells. The CYP expression level in the human small intestine is reported to be approximately 80% for CYP3A4, approximately 15% for CYP2C9, approximately 2% for CYP2C19, and approximately 1% for CYP2D6 [20]. It will be necessary to evaluate whether the proportion of CYP expression in the Caco-2 4CYPs-MAC #2 is close to that of the human small intestine.

If CYP metabolic capacity is guaranteed in the established clones, the established cell line can be used as new human small intestine model cells. In recent years, small intestine model cells prepared from induced pluripotent stem cells have been reported, but such cells are considered difficult to use for screening large quantities of drug candidate compounds [21]. However, Caco-2 cells are easy to handle. Therefore, it is possible to use CYP-modified Caco-2 cells to test large numbers of candidate compounds as a first screening.

## Conclusion

The mammalian artificial chromosome vector system would provide useful models for drug development. The established Caco-2 cells with the 4CYPs-MAC are expected to more accurately predict absorption and metabolism in the human intestine than parental Caco-2 cells.

## Methods

### Cell culture

Chinese hamster ovary (CHO) cells (JCRB0218, JCRB Cell Bank, NIBIOHN, Osaka, Japan) carrying the 4CYPs-MAC were maintained in Ham’s F-12 nutrient mixture (Wako, Osaka, Japan) supplemented with 10% fetal bovine serum (FBS) and 600 μg/mL G418. Parental Caco-2 cells (ATCC® HTB-37™, ATCC, Manassas, VA, USA) were maintained in Dulbecco’s modified Eagle’s medium (DMEM) (Wako) supplemented with 10% FBS, MEM non-essential amino acids (Gibco, Thermo Fisher Scientific, Waltham, MA, USA), 1 M HEPES (Gibco), 100 mM sodium pyruvate (Gibco), 200 mM GlutaMAX (Gibco), and penicillin-streptomycin (Wako). Caco-2 cells with the 4CYPs-MAC were maintained in the above medium supplemented with 250 μg/mL G418. These cells were cultured at 37 °C in 5% CO_2_.

### Microcell-mediated chromosome transfer

Transfer of 4CYPs-MAC from CHO cells to Caco-2 cells was performed using a standard procedure [22]. Briefly, donor CHO cells were cultured in F12 medium supplemented with 20% FBS and 0.1 μg/mL colcemid. After 72 h, microcells were isolated through centrifugation with DMEM containing cytochalasin B and filtration. Then, microcells suspended in phytohemagglutinin P (PHA-P)/DMEM were poured onto Caco-2 cells in a 6-cm dish and incubated for 15 min. The cells were treated with polyethylene glycol (PEG) solution (5 g of PEG1000, 6 mL of DMEM, 1 mL of dimethyl sulfoxide) for 1 min followed by washing with DMEM. After 24 h of recovery culture, cells were seeded in a 24-well collagen-coated plate (Corning, NY, USA) and maintained with selection medium 24 h after seeding. Thereafter, the medium was changed twice a week to obtain drug-resistant clones. Because the MAC carries a GFP gene, GFP-positive clones were selected from the drug-resistant clones.

### Genomic PCR analyses

We extracted genomic DNA from cell lines using a genomic DNA extraction kit with DNase-free RNase (Gentra Systems, Minneapolis, MN, USA). The primers for the genomic PCR are listed in Additional file 1: Table S1. They amplified each gene region on the 4CYPs-MAC. We used KOD FX (Takara, Otsu, Japan) in accordance with the manufacturer’s instructions.

### FISH analyses

Trypsinized cells were incubated for 15 min in 0.075 M KCl and fixed with methanol and acetic acid (3:1), and then slides were prepared using standard methods. FISH analyses were performed using the fixed metaphase of each cell hybrid using digoxigenin-labeled (Roche, Germany) DNA [Mouse Cot-1 DNA (Invitrogen, Carlsbad, CA, USA)] and biotin-labeled DNA [PAC 4CYPs-POR], essentially as described previously [23]. Chromosomal DNA was counterstained using DAPI (Sigma-Aldrich, St. Louis, MO, USA). Images were captured using an AxioImagerZ2 fluorescence microscope (Carl Zeiss, Germany).

### RT-qPCR

We extracted mRNA using the RNeasy Mini Kit (Qiagen, Germany) and synthesized first-strand cDNA using the High Capacity cDNA Reverse Transcription Kit (Applied Biosystems, Foster City, CA, USA). The primers are listed in Additional file 1: Table S1. For RT-qPCR analysis, TB Green Premix Ex Taq (Takara) was used and relative mRNA expression was evaluated through the ΔΔCt method. GAPDH was used for normalization.

### Culture time-dependent expression level change of four CYPs

Caco-2 cells and Caco-2 4CYPs-MAC #2 were seeded in a 6-cm dish at a concentration of 7.0 × 10^5^ cells/well. Cells were lysed using Trizol (Invitrogen, CA, USA) on days 4, 11, and 22 after seeding, and RNA was extracted and purified using an RNeasy Mini Kit (Qiagen). Thereafter, cDNA synthesis was performed using the High-Capacity cDNA Reverse Transcription Kit (Applied Biosystems).

### Activity test of the four CYPs

We tested the metabolic activity of the four CYPs using the P450-Glo™ assay (Promega, Madison, WI, USA). The luminogenic substrates used for the test were Luciferin-IPA (CYP3A4), Luciferin-ME EGE (CYP2D6), Luciferin-H (CYP2C9), and Luciferin-H EGE (CYP2C19). Cells were seeded in 48-well collagen-coated plates (Corning) at a density of 1 × 10^4^ cells/well. After 48 h, the medium was changed and 72 h later we added transport medium (TM) containing substrate. TM was prepared using Hanks’ Balanced Salt Solution (HBSS) with 4.2 mM NaHCO_3_, 20 mM glucose, and 10 mM HEPES, which was adjusted to pH 7.4. After incubation, we added detection reagent and measured the luminescence using an Infinite F500 plate reader (Wako). CYP2C9 and CYP2C19 required 4 h of incubation, while CYP2D6 and CYP3A4 required 1 h. After the measurement, the cells were washed with PBS, dissolved in lysis buffer, and diluted fivefold. The same amount of Cell Titer Glo (Promega) was added to 10 μL of the lysate, and luminescence measurement was performed to normalize data to the number of viable cells.

### Midazolam (MDZ) permeability test

The obtained clones were assessed in an MDZ permeation test. Each cell was seeded on a 24-well cell culture insert plate (Millipore, Billerica, MA, USA) at a concentration of 2.5 × 10^4^ cells/well. The medium was changed once a week after seeding and every 2 d after the second week. Transepithelial electrical resistance (TEER) was measured using Millicell-ERS (Millipore) before medium exchange. The test was performed 23 d after seeding. For the test, TM (pH 7.4) prepared by adding 4.2 mM NaHCO_3_, 20 mM glucose, and 10 mM HEPES to HBSS at pH 7.4 was used. The donor side solution was prepared by dissolving 3 μM MDZ in TM (pH 6.4). The acceptor side solution was prepared by adding 4.5% FBS to TM (pH 7.4). On the test day, the medium was removed from the culture insert seeded with the cells and the cells were rinsed twice with TM (pH 7.4). TM (pH 6.5) and TM (pH 7.4) were added to the apical and basal chambers, respectively, and cells were incubated at 37 °C for 30 min. The test was started by adding the donor side solution to the donor side chamber and the acceptor side solution to the acceptor side chamber. Thirty minutes after the start of the test, the solution in the donor side and acceptor side chambers was collected. Moreover, to measure the amount of MDZ and 1′-hydroxy MDZ (1′-OH MDZ) in the cells, after the test the culture insert was quickly rinsed three times with ice-cold TM (pH 7.4). The membrane was cut using a cutter, and 70 μL of ice-cold TM (pH 7.4) was added. Cells were detached from the membrane through sonication and a cell suspension was used as a sample. These samples were deproteinized and stored at − 80 °C until measurement.

LC-MS/MS was used for the measurement of MDZ and 1′-OH MDZ in the samples. QTRAP5500 (SCIEX, Framingham, MA, USA) and a Prominence UFLC system (Shimadzu, Kyoto, Japan) were combined for measurement. The HPLC conditions and MS/MS conditions are shown in Additional file 1: Table S2. Quantitative analysis was performed in multiple reaction monitoring mode. Mass transitions (m/z) were 326.1 → 291.1 for MDZ, 342.1 → 168.0 for 1′-OH MDZ, and 346.1 → 169.2 for 1′-OH MDZ d4. Data were analyzed using Analyst 1.5 software (SCIEX).

Equation 1 Formula to calculate extraction ratio (ER)

$$ \mathrm{ER}=\frac{\sum {\mathrm{metabolite}}_{\left(\mathrm{donor}+\mathrm{receiver}+\mathrm{intracellular}\right)}}{{\mathrm{parent}}_{\left(\mathrm{receiver}+\mathrm{intracellular}\right)}+\sum {\mathrm{metabolite}}_{\left(\mathrm{donor}+\mathrm{receiver}+\mathrm{intracellular}\right)}} $$

### Inhibition test

Ketoconazole (Tokyo Chemical Industry, Tokyo, Japan) was used as an inhibitor against CYP3A4 and changes in metabolic activity were measured using a P450-Glo assay with luciferin-IPA. Cells were seeded in a 48-well collagen-coated plate at 2.5 × 10^4^ cells/well, and the medium was changed after 2 d. The next day, the medium was collected, cells were washed twice with PBS, and then 100 μL of TM (pH 6.5) containing ketoconazole (Tokyo Chemical Industry) at 0, 0.01, 0.1, 1.0, 10, and 100 μM was added to each set of three wells. TM was adjusted to pH 6.5 by adding 4.2 mM NaHCO_3_, 20 mM glucose, and 10 mM MES to HBSS. The cells were preincubated for 1 h at 37 °C and 1000-fold diluted CYP3A4 substrate was added. One hour later, 50 μL of the supernatant was collected from the well, mixed with 50 μL of detection reagent, and the luminescence value was measured using an Infinite F500 plate reader (Wako).

## Supplementary information

**Additional file 1: Figure S1.** Comparison of gene expression between human small intestine and day 22 culture of Caco-2 and Caco-2 4CYPs-MAC #2. **Table S1.** Primer sequences for genomic PCR and RT-qPCR. **Table S2.** LC-MS/MS analysis conditions (MDZ, 1′-OH MDZ).

## Data Availability

The data and materials used and/or analyzed during the current study are available from the corresponding author on reasonable request.

## Abbreviations

CYP: Cytochrome P450
AC: Artificial chromosome
MMCT: Microcell-mediated chromosome transfer
POR: P450 oxidoreductase
HAC: Human artificial chromosome
MAC: Mouse artificial chromosome
DDI: Drug–drug interaction
CHO: Chinese hamster ovary
MDZ: Midazolam
TEER: Transepithelial electrical resistance
ER: Extraction ratio
