# Supplementary material for: Development of Caco-2 cells expressing four CYPs via a mammalian artificial chromosome
Source: BMC Biotechnol. 2020 Aug 20;20:44. doi: 10.1186/s12896-020-00637-8 (PMC7441628; doi:10.1186/s12896-020-00637-8)
Supplement: Supplementary file 1 — Additional file 1: Figure S1. Comparison of gene expression between human small intestine and day 22 culture of Caco-2 and Caco-2 4CYPs-MAC #2. Table S1. Primer sequences for genomic PCR and RT-qPCR. Table S2. LC-MS/MS analysis conditions (MDZ, 1′-OH MDZ). [file 12896_2020_637_MOESM1_ESM.pdf]

Supplementary material

Title: Development of Caco-2 cells expressing four CYPs via a mammalian artificial chromosome

Yumi Ohta[1], Kanako Kazuki[2], Satoshi Abe[3], Mitsuo Oshimura[3], Kaoru Kobayashi[4], Yasuhiro Kazuki[1,2,*]

1. Division of Genome and Cellular Functions, Department of Molecular and Cellular Biology, School of Life Science, Faculty of Medicine, Tottori University, 86 Nishi-cho, Yonago, Tottori 683-8503, Japan

2. Chromosome Engineering Research Center (CERC), Tottori University, 86 Nishi-cho, Yonago, Tottori 683-8503, Japan

3. Trans Chromosomics, Inc., 86 Nishi-cho, Yonago, Tottori 683-8503, Japan

4. Laboratory of Biopharmaceutics, Meiji Pharmaceutical University, 2-522-1 Noshio, Kiyose, Tokyo 204-8588, Japan

*Correspondence should be addressed to Y.K. (kazuki@tottori-u.ac.jp).

Division of Genome and Cellular Functions, Department of Molecular and Cellular Biology, School of Life Science, Faculty of Medicine, Tottori University, 86 Nishi-cho, Yonago, Tottori 683-8503, Japan. Tel: +81-859-38-6219. Fax: +81-859-38-6210.

Supplementary Figure

Figure S1. Comparison of gene expression between human small intestine and day 22 culture of Caco-2 and Caco-2 4CYPs-MAC #2.

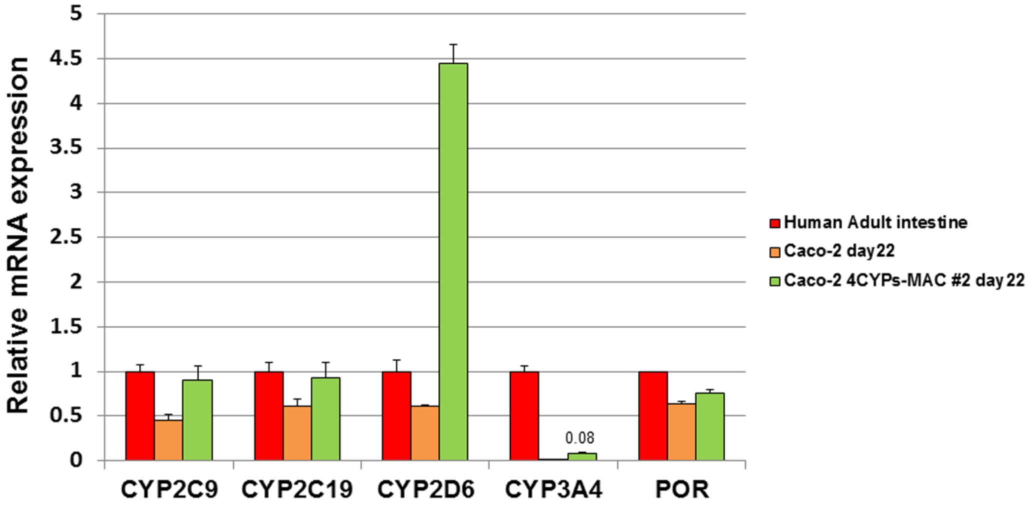

Supplementary Tables

Table S1. Primer sequences for genomic PCR and RT-qPCR

Aim	Target gene	Primer name	Sequence (5'-3')
gPCR	CYP2C9	gPCR hCYP2C9_F	ATGGATTCTCTTGTGGTCCT
		gPCR hCYP2C9_R	TCAGACAGGAATGAAGCACA
	CYP2C19	qPCR hCYP2C19_F	ACTTGGAGCTGGGACAGAGA
		CAGpA_rev1764	CAGCCACCACCTTCTGATAGG
	CYP2D6	qPCR hCYP2D6_F	TGATGAGAACCTGCGCATAG
		CAGpA_rev1801	CTCAGTGGTATTTGTGAGCC
	CYP3A4	qPCR hCYP3A4_F	TGTGGGGCTTTTATGATGGT
		CAGpA_rev1801	CTCAGTGGTATTTGTGAGCC
	POR	qPCR hPOR_F	TGGAGGAGGACTTCATCACC
		CAGpA_rev1801	CTCAGTGGTATTTGTGAGCC
RT-qPCR	CYP2C9	qPCR hCYP2C9_F	CCACATGCCCTACACAGATG
		qPCR hCYP2C9_R	TGCCCTTGGGAATGAGATAG
	CYP2C19	qPCR hCYP2C19_F	ACTTGGAGCTGGGACAGAGA
		qPCR hCYP2C19_R	CATCTGTGTAGGGCATGTGG
	CYP2D6	qPCR hCYP2D6_F	TGATGAGAACCTGCGCATAG
		qPCR hCYP2D6_R	CCCTATCACGTCGTCGATCT
	CYP3A4	qPCR hCYP3A4_F	TGTGGGGCTTTTATGATGGT
		qPCR hCYP3A4_R	CCTCCGGTTTGTGAAGACAG
	POR	qPCR hPOR_F	TGGAGGAGGACTTCATCACC
		qPCR hPOR_R	ACAAGCTCGTACTGGCGAAT
	GAPDH	GAPDH real F	AGCCACATCGCTCAGACAC
		GAPDH real R	GCCCAATACGACCAAATCC

Table S2. LC-MS/MS analysis conditions (MDZ, 1’-OH MDZ)

HPLC condition	
column	TSK gel ODS-100V column (50 mm x 0.2 mm, 3µm, TOSOH)
mobile phase	A: 0.1% formic acid prepared with MQ
	B:0.1% formic acid prepared with acetonitrile
gradient condition	A:B=80:20 (v/v)
flow velocity	0.2ml/minute
column temperature	40℃
injection amount	10µl

MS/MS condition	
Ionization method	ESI+
curtain gas1	30psi
curtain gas2	40psi
collision gas	9
Ionspray voltage	5500V
Declustering Potential (DP)	100V
Entrance potential	10V
Collision energy	40V
Collision cell exit potential	10V
Ion source temp	650℃
